# 1821–2021: Contributions of physicians and researchers of Greek descent in the advancement of clinical and experimental cardiology and cardiac surgery

**DOI:** 10.3389/fcvm.2023.1231762

**Published:** 2023-08-04

**Authors:** Apostolos Gerontas, Dimitrios Avgerinos, Konstantinos Charitakis, Helena Maragou, Konstantinos Drosatos

**Affiliations:** ^1^ School of Applied Natural Sciences, Coburg University, Coburg, Germany; ^2^ School of Liberal Arts and Sciences, The American College of Greece, Athens, Greece; ^3^ Department of Cardiac Surgery, Onassis Cardiac Surgery Center, Athens, Greece; ^4^ ARISTEiA-Institute for the Advancement of Research and Education in Arts, Sciences and Technology, McLean, VA, United States; ^5^ Department of Internal Medicine, Division of Cardiology, University of Texas Health Science Center, Houston, TX, United States; ^6^ Metabolic Biology Laboratory, Cardiovascular Center, Department of Pharmacology and Systems Physiology, University of Cincinnati College of Medicine, Cincinnati, OH, United States

**Keywords:** Greece, cardiology, cardiac surgery, cardiovascular research, pioneers, experimental cardiology, physicians, researchers

## Abstract

While the role of Greeks in the development of early western medicine is well-known and appreciated, the contributions of modern Greek medical practitioners are less known and often overlooked. On the occasion of the 200-year anniversary of the Greek War of Independence, this review article sheds light onto the achievements of modern scientists of Greek descent in the development of cardiology, cardiac surgery, and cardiovascular research, through a short history of the development of these fields and of the related institutions in Greece. In the last decades, the Greek cardiology and Cardiac Surgery communities have been active inside and outside Greece and have a remarkable presence internationally, particularly in the United States. This article highlights the ways in which Greek cardiology and cardiovascular research has been enriched by absorbing knowledge produced in international medical centers, academic institutes and pharmaceutical industries in which generations of Greek doctors and researchers trained prior to their return to the homeland; it also highlights the achievements of medical practitioners and researchers of Greek descent who excelled abroad, producing ground-breaking work that has left a permanent imprint on global medicine.

## Introduction

Early studies on the function and diseases of the heart can be traced to the classical antiquity, with Hippocrates's pioneer anatomical studies setting the stage for future developments (1). However, it took over a millennium for the English physician William Harvey (1578–1657) to set the foundation of cardiology by introducing a daring model for blood circulation in 1628. This model disproved the previously accepted ebb-and-flow theory and showed the heart to function as a pump in the center of the circulatory system. A little later, the English physician, Richard Lower (1631–1691), took a step further by proposing that blood circulation's primal purpose was to serve the exchange between air and blood (2). The work of the Greek physician, Alexandros Mavrokordatos (1636–1709), constituted a significant contribution in that direction. In 1664, Mavrokordatos presented experimental evidence on animals showing that blood circulates in the lungs (3). And yet, despite these promising beginnings, cardiology did not show remarkable development before the emergence of breakthrough research in the 20th century.

In the 20th century, new diagnostic tools allowed studies in living organisms, which expedited the understanding of cardiac function in health and disease. Modern studies of the heart, as well as development of relevant technological methods and tools, were instrumental in effecting a separation of cardiology from general internal medicine; in this context, it is worth noting the development of electrocardiography during the first decade of the 20th century, of catheterization in the 1930 s, of echocardiography in the 1950 s, as well as of radiological studies that emerged during these decades. Advancements in diagnostic methods were accompanied by deployment of technological and pharmaceutical solutions and eventually by advanced surgical and minimally invasive interventional methods. Cardiothoracic surgery was greatly developed when John Heysham Gibbon (1903–1973) invented the cardiopulmonary bypass machine in 1953. The discovery of the DNA structure in 1953, combined with major advancements in molecular biology and genetics over the last 70 years, proved game-changers in improving understanding of cardiovascular diseases.

Despite systemic difficulties that periodically impeded progress, cardiology, cardiac surgery and research in modern-day Greece reflected the European trends in both research and practice, particularly those of Germany and France. A first generation of cardiologists existed already from the 1930 s in hospital clinics in Athens and Thessaloniki. During these early years, cardiologists were in fact internal medicine experts who specialized in heart problems, as cardiology did not achieve the status of an independent discipline till the late 1940 s. After the Second World War, Greek cardiology received most of its influences from researchers and practitioners in the US, rather than Germany and France.

## Cardiology in Greece

**Figure 1:**
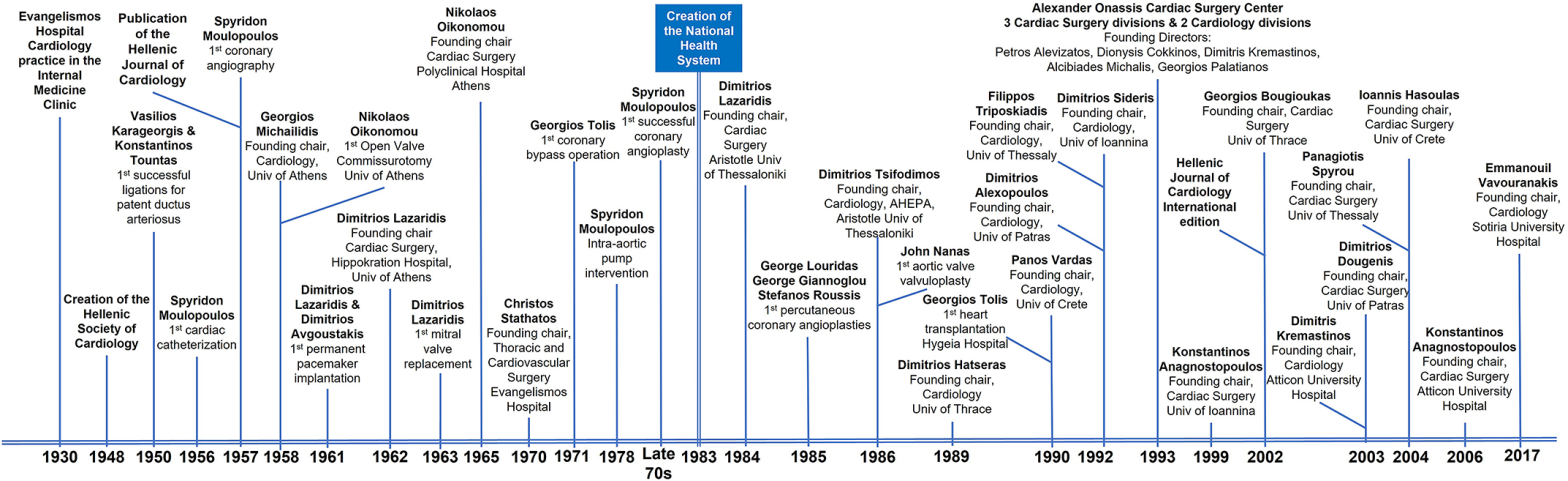
Important events and contributors in the development of cardiology and cardiac surgery in Greece.

While the idea of cardiology as a separate discipline had matured in Greece for some years prior to the Second World War, the war delayed its realization until 1947 (Figure 1), when American physicians, led by the prominent cardiologist Paul Dudley White (1886–1973) brought to Greece the first modern ECG system. A few months later, in December 1948, the Hellenic Society of Cardiology came into being. This took place during an unofficial meeting of approximately 30 cardiologists in Athens at the house of Demosthenes Papapanagiotou. The Society was officially registered in 1950, and cardiology broke away from internal medicine to become a distinct field of specialization in 1954.

In 1958, the University of Athens announced the first chair of cardiology, a position filled by Georgios Michailidis (1906–1974) in 1961, who also served as the president of the Hellenic Society of Cardiology. Georgios Michailidis, a man with a very dynamic personality and educated in Paris, left a lasting influence on the field of cardiology in Greece. Over the years, several other prominent cardiologists served as presidents of the Society (Table 1). The Society launched publication of its journal in 1957 (it is worth noting that since 2002, *The Hellenic Journal of Cardiology* has also an international publication, largely through the efforts of Panos Vardas).

**Table 1:** Presidents of the hellenic society of cardiology.

Years	HSC president	Years	HSC president
1950–1951	Konstantinos Maroulis	1990–1991	Dionysios Cokkinos
1952	Panagiotis Vatsineas	1992–1993	Ioannis Gialaphos
1953	Nikolaos Tsouchlos	1994–1995	Apostolos Zacharoulis
1955–1956	Dimitrios Papapanagiotou	1996–1997	Evangelos Papasteriades
1959	Gerasimos Danilatos	1998–1999	Christodoulos Stefanadis
1960 & 1965–1966	Anastasios Samaras	2000–2001	Panagiotis Vardas
1961–1962 & 1967–1968	Georgios Michailidis	2002–2003	Dimitris Sideris
1969–1970	Christos Aravanis	2004–2005	Ioannis Lekakis
1971–1972	Phroixos Kosteas	2006–2007	Charisios Mpountoulas
1973–1974	Eftychios Voridis	2007–2008	Dimitrios Kremastinos
1975–1976	Spyridon Moulopoulos	2009–2010	Vlassios Pyrgakis
1977–1978	Meletios Pappas	2011–2012	Georgios Parcharidis
1979–1981	Dimitrios Athanasiadis	2013–2014	Ioannis Kallikazaros
1982–1983	Georgios Pipilis	2015–2016	Stephanos Phoussas
1984–1985	Dimitrios Avgoustakis	2017–2018	Konstantinos Tsioufis
1986–1987	Pavlos Toutouzas	2019–2020	Ioannis Goudevenos
1988–1989	Lampros Anthopoulos	2021–2022	Ioannis-Georgios Kanakakis

Georgios Michailidis served also as the founding director of the 1st University Cardiology Clinic at the Hippokration University Hospital, between 1961 and 1971. He was succeeded by Dimitrios Avgoustakis (1971–1984), Pavlos Toutouzas (1984–2003), Christodoulos Stefanadis (2003–2014), Dimitrios Tousoulis (2014–2020), and Konstantinos Tsioufis (2020 -). This clinic has been pivotal to the establishment of the field and its expansion. The contributions of one of the directors, Pavlos Toutouzas, were essential for the establishment of the catheterization lab of the clinic and cardiology training, as well as to the diffusion of cardiology-related knowledge to the public.

Other developments in Greek cardiology from the 1950s are associated with Spyridon Moulopoulos, one of the most active cardiology researchers. Moulopoulos was based in Greece but his work had an international impact. In 1956, he performed the first catheterization and in 1957 the first coronary angiography in the country, adding his name among pioneers from abroad (4). Later, in the USA, he undertook research on the treatment of end-stage heart failure and was one of the main contributors to the invention of the intra-aortic balloon pump (5, 6), which was the only interventional treatment for cardiogenic shock for over 40 years.

In 2003, the newly constructed public “*Atticon”* University Hospital established its cardiology unit under the leadership of Dimitris Kremastinos, who was a member of the European Academy of Sciences and Arts and an influential figure of Greece, having served as the Minister of Health and the chief physician of Prime Minister Andreas Papandreou.

The most recently established academic unit of cardiology is the one at the “Sotiria” University Hospital in Athens, which operates under the directorship of Emmanouil Vavouranakis since 2017. This is the “3rd Cardiology Clinic”, which was originally founded by Ioannis Nanas in 2002, at the Laikon University Hospital of Athens.

Still, cardiology as a field of academic research remained confined within Athens until the late 1980s and the early 1990s. Clinical divisions of cardiology were eventually established at the Democritus University of Thrace (officially founded in 1987, but fully functional after 1989, under the directorship of Dimitrios Hatseras), at the University of Crete in Heraklion in 1990 (under the leadership of Panos Vardas), while 1992 witnessed the simultaneous founding of clinical divisions at the University of Patras (under Dimitrios Alexopoulos), at the University of Ioannina (under Dimitrios Sideris), and at the University of Thessaly (under Filippos Triposkiadis).

## Cardiac surgery in Greece

Cardiac surgery in Greece had an impressively early start (Figure 1) -for a small country recovering from World War II, the German occupation, and civil war—especially considering that this start preceded the key event of John Gibbon's ground-breaking application of the cardiopulmonary bypass system in 1953. In 1950, Vasilios Karageorgis and Konstantinos Tountas performed independently the first successful ligation procedures for patent ductus arteriosus.

The 1960 s were a revolutionary period for several medical fields, due to medical technology advances, and Greek cardiac surgery followed international developments. The first implantation of a permanent pacemaker was conducted in 1961 by Dimitrios Lazaridis and Dimitrios Avgoustakis; two years later, Lazaridis performed successfully the first mitral valve replacement. During this period, Konstantinos Tountas organized the first training demonstrations in cardiac surgery at the *American Hellenic Educational Progressive Association* (*AHEPA*) hospital in Thessaloniki; in 1962, Dimitrios Lazaridis founded the first dedicated cardiac surgery unit at the Hippokration hospital in Athens and was succeeded by Georgios Andritsakis in 1965. Concurrently, one more clinical team, under Nikolaos Oikonomou, was operating at the *Polyclinic Hospital of Athens* (7).

The Athens hospital of *Evangelismos* was operating a cardiological practice as part of the Internal Medicine clinic since the 1930 s. The hospital introduced cardiac surgery in the 1970 s, with the founding of the Department of Thoracic and Cardiovascular Surgery by Christos Stathatos, and expanded through the pioneering work of George Tolis—the first United States (US)-trained specialist in cardiothoracic surgery in Greece. This unit has played an important role in the establishment and development of the field in Greece. Georgios Tolis performed the first coronary bypass operation in Greece in 1971.

Cardiology and cardiac surgery have been revolutionized by the development of minimally invasive interventional methods, which were adopted in the 1930 s and gained in popularity during the 1940 s and 1950 s. As we have seen, the therapeutic clinic of the University of Athens, under the leadership of Spyridon Moulopoulos, had been performing catheterizations and coronary angiographies since the late 1950 s. The application of the intra-aortic pump, to the development of which Moulopoulos had contributed while in the US, was performed in 1978. These initiatives, along with the efforts of the Cardiology Clinic of *Hippokration*, directed by Dimitrios Avgoustakis, who built upon the work of Dimitrios Lazaridis and his successors, contributed to the evolution of cardiac surgery. The late 1970 s witnessed Greek cardiology reaching the milestone of the successful application of coronary angioplasty.

Changes in the Greek health care system as a result of the creation of the public National Health System (NHS/“*ΕΣΥ*”) in 1983, impacted cardiology and cardiac surgery in Greece. The previously existing units were incorporated into the NHS, which precipitated major organizational restructuring and leadership changes. In the aftermath of the reform, the *Evangelismos* Hospital of Athens developed two different cardiac surgery departments led by Fotis Panagopoulos and Christos Lolas respectively. The *Hippokration* Hospital operated three departments led by Andreas Vomboyiannis, Emmanouil Chlapoutakis, and Ioannis Papaioannou. In the “*Agia Sophia” Children's Hospital* there were two departments of cardiac surgery for children, led by Georgios Andritsakis and Ioannis Stinios. Georgios Sanoudos was assigned as the director of the “*417 Army Equity Fund” Hospital* cardiac surgery unit. The cardiac surgery units of *George Papanikolaou Hospital* and the *AHEPA* hospital in Thessaloniki were led by Panagiotis Spyrou and Dimitrios Lazaridis, respectively. The new leaders were entrusted with the reorganization of the clinics for them to serve both educational and healthcare responsibilities. In 1985, Stefanos Roussis, Georgios Louridas, and Georgios Giannoglou performed the first percutaneous coronary angioplasties in Greece at the Papanikolaou Hospital and the AHEPA Hospital of Thessaloniki.

The first heart transplantation in the country was done by George Tolis in 1990, at the Hygeia Therapeutic Center. Later, as the demand for specialized cardiac surgery care started to rise, Medical Schools of Greek universities outside of Athens and Thessaloniki responded by creating specialized units. Such units were founded at the University of Ioannina in 1999 under Konstantinos Anagnostopoulos, at the Democritus University of Thrace in 2002 under Georgios Bougioukas, at the University of Patras in 2003 led by Dimitrios Dougenis, at the University of Thessaly in 2004 under Panagiotis Spyrou, and at the University of Crete in 2004 under Ioannis Hasoulas. In 2006, the newly constructed public “*Atticon” University Hospital* established its first cardiac surgery unit under the leadership of Konstantinos Anagnostopoulos. This unit was added to the cardiology unit functioning there since 2003.

An important development for cardiac surgery in Greece was the opening of a fully dedicated cardiac surgery center that was funded by and named after the “*Alexandros Onassis” Foundation.* The *Onassis Cardiac Surgery Center* (known as *Onassion*) started operating in 1993. A unique institution displaying a high level of specialization, *Onassion* became equipped with cutting-edge technologies and was manned by internationally acknowledged experts from the outset. Soon, it was in a position to respond to a great percentage of the cardiac surgery needs of the country. The first surgeons who staffed Onassion were Alcibiades Michalis, Georgios Palatianos, Petros Alevizatos, Georgios Economopoulos and Konstantinos Anagnostopoulos. Petros Alevizatos performed the first heart transplantation surgery at Onassion. The Onassis Cardiac Surgery Center included two cardiology clinics in its operational structure. Dionysis Cokkinos was the director of the First Cardiology Clinic of the Hospital from the year of its foundation. He ran both clinical service and research that focused on the study of preconditioning in diseased hearts. Dimitris Kremastinos directed the Second Cardiology Clinic.

## Cardiovascular research

**Figure 2:**
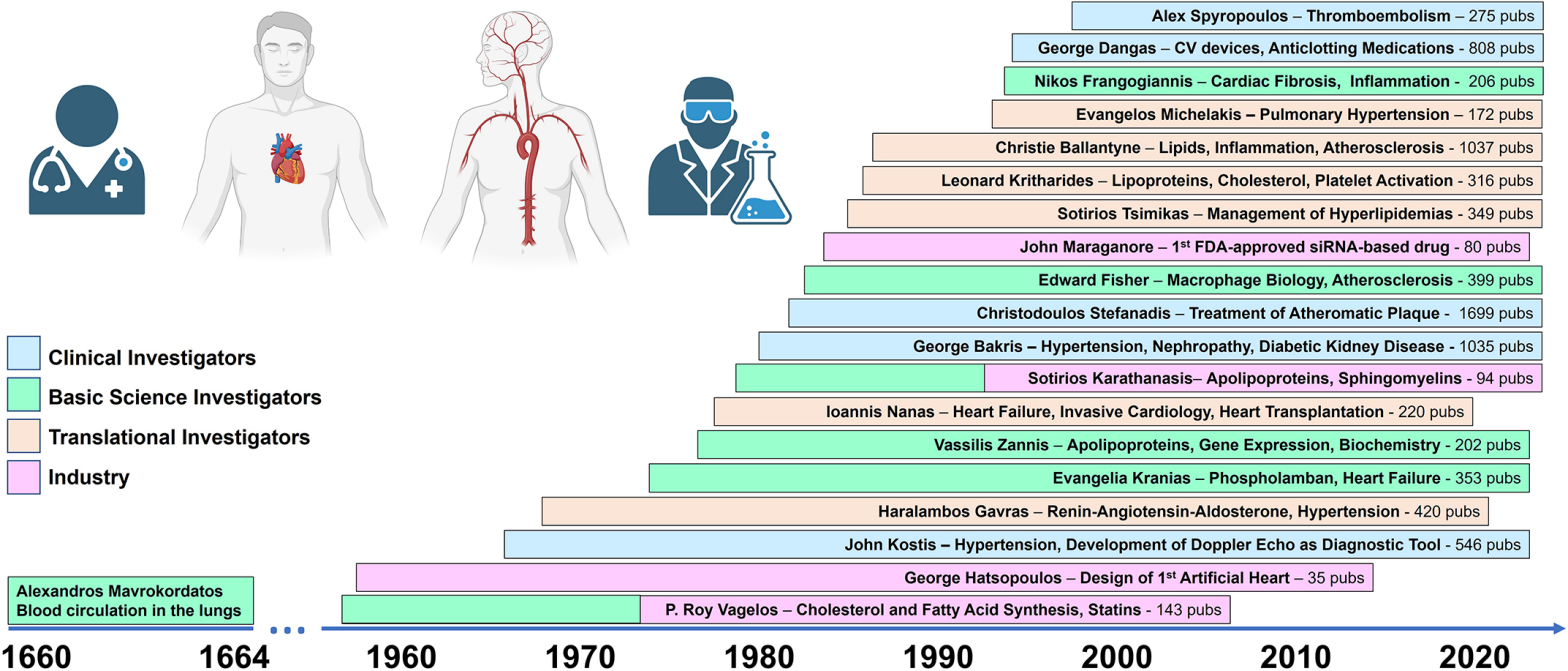
Cardiovascular research pioneers of Greek descent. Timelines indicate periods of active research productivity documented with publication of research articles. Publication records were extracted from the Scopus Database. Graphics were made with biorender.com.

Following the establishment of the cardiology and cardiac surgery clinical practice in Greece, graduates of medical schools and biomedical departments of Greek universities started exploring opportunities for cardiovascular research and more advanced clinical training in Greece and abroad. Particularly, in the last 30 years, it has become evident that as in many medical disciplines, the development of drugs for cardiovascular diseases requires the active cooperation of basic science researchers, clinical investigators, and pharmaceutical industry experts. Scientists of Greek descent have contributed important findings in all three platforms of the drug discovery and development arena (Figure 2).

Below are profiles of eminent scientists of Greek descent who contributed to the field of cardiology and cardiac surgery, both internationally and within Greece.

### Heart failure

Evangelia Kranias, one of the few Greek women who conduct basic cardiovascular research, identified the importance of Phospholamban (PLN) in the regulation of calcium cycling through the sarcoplasmic reticulum and the overall regulation of cardiac function (8–10). Her team was also instrumental in demonstrating the clinical relevance of PLN in heart failure and identifying human mutations in this gene that result in heart failure and/or arrhythmias (11). A specific mutation characterized by loss of arginine (PLN-R14del) was originally discovered in Greece but subsequently found in multiple families in Europe, Canada, and the US (12). The patients have formed the PLN Foundation, which has grown into a large organization and an international consortium of basic and clinical investigators and patient representatives has been formed. Kranias coordinates the US efforts to better understand the arrhythmogenic cardiomyopathy caused by the PLN-R14del mutation and to develop effective treatments. Her contributions starting from studies in cells and animals and exploring clinical applications constitute one of the rare examples of people who have devoted their entire life to elucidate the function of a single protein and apply the knowledge for treating a disease. Thus, although the exploration for PLN-based therapeutics is still in progress, the research community has designated Evangelia Kranias as the “mother of phospholamban”.

Ioannis (John) Nanas was a pioneer of invasive cardiology and heart transplantation, as well as an innovator in establishing clinical practice and education programs in Greece. He created an international training network for Greek physicians, organized around the University of Athens in Greece and the University of Utah in the USA. This network was instrumental in establishing the first Heart Failure Program (1980's), the first Heart Transplantation Program (1990's), the first durable Mechanical Circulatory Support program (2000's), and the first Primary Percutaneous Coronary Intervention program for acute myocardial infarction in Greece. Besides performing the first aortic valve valvuloplasty in the country in 1986, he directed an expanded research program that has impacted daily clinical practice around the globe. The innovative experimental research performed by his group on the efficacy of amiodarone on ventricular arrhythmias set the foundation for later clinical trials that identified the value of using amiodarone for cardiopulmonary resuscitation (CPR)/cardiac arrest therapeutic algorithm (13, 14). In the field of heart failure (HF), he led a multicenter randomized clinical study, performed in Greece and found no significant benefit from using higher than the standard dose of enalapril. This trial led to the establishment of the optimal dose of ACE-inhibitors in the international heart failure guidelines (15). His research was also crucial for identifying iron deficiency as the most frequent cause of anaemia in chronic heart failure instead of describing it as “anaemia of chronic disease”. This finding led to new therapies for anaemia in chronic heart failure (16). Furthermore, he was the inventor of the para-aortic counterpulsation device which is an implantable system that stabilizes decompensated heart failure and cardiogenic shock patients (17, 18).

Having completed his medical studies in Greece, Nikolaos Frangogiannis moved to the US for clinical training and research. His research examines the cellular and molecular mechanisms of myocardial inflammation and fibrosis in acute myocardial infarction and chronic heart failure. Early work from his laboratory has demonstrated that chemokines (such as CCL2/MCP−1) and cytokines (such as IL-b and TNF-a) are central mediators in the repair and remodeling of the infarcted heart (19, 20). His work has also dissected the molecular signals and cellular effectors involved in the suppression and resolution of the post-infarction inflammatory response. His more recent work has revealed critical cell-specific action of TGF-β signaling cascades in cardiac remodeling (21, 22) has unraveled the complex interactions between structural and matricellular components of the extracellular matrix network and has dissected cellular mechanisms of cardiac fibrosis in response to ischemia, mechanical stimulation or metabolic overload. Overall, Nikolaos Frangogiannis has been among the leading investigators who have contributed to better understanding of the molecular mechanisms that underlie two of the most critical conditions for heart failure: inflammation and fibrosis.

### Vascular biology

One of the early contributors to molecular studies of lipoprotein metabolism was Vassilis I. Zannis, who for more than 25 years divided his academic work between Boston University in the US and the University of Crete, Greece. He served as the founding director of the Division of Molecular Genetics at the Medical School of Boston University, and since 1985 as professor of Biochemistry at the newly established medical school of the University of Crete. The work of his laboratories in the two countries focused on the characterization of the genes and structure of Apolipoproteins, their transcriptional regulation (23), and their involvement in lipoprotein metabolism disorders that lead to hyperlipidemia and cardiovascular disease (24). One of the most historical discoveries of Vassilis Zannis was the identification of the 3 isoforms of human Apolipoprotein E, ApoE2, ApoE3, and ApoE4 (25, 26). This discovery set the foundation for the future association of ApoE2 with cardiovascular disease and of ApoE4 with Alzheimer's Disease, which allows early identification -with genetic screening- of people that are of high risk for developing these diseases. He also contributed to research about HDL biogenesis and Very Low-Density Lipoprotein (VLDL) clearance by identifying the role and critical domains of ApoE (27, 28) and ApoA-I (29) in these processes that are important for systemic lipid homeostasis and understanding of the process that leads to atherosclerosis.

Edward A. Fisher, whose mother was born in Thessaloniki and moved with her family to the US, has made important contributions to cardiovascular disease research in 3 areas: his lab and collaborators discovered the major pathways regulating Apolipoprotein B availability for its assembly into VLDL particles, the precursors of LDL, including the regulation of these pathways by fatty acids, including those enriched in the Mediterranean diet (30). Furthermore, his teams established robust models of the regression of atherosclerotic plaques, which showed, among other discoveries, that LDL lowering was more effective in regression when plaque inflammation was concomitantly reduced, anticipating the findings of the landmark CANTOS clinical trial (31). His lab adapted High-Density Lipoprotein (HDL) particles to deliver different cargoes to macrophages in atherosclerotic plaques, and in pre-clinical studies with the groups of Zahi Fayad and Wilhem Mulder at Mount Sinai School of Medicine, they have shown these nanoparticles to have theranostic potential- i.e., useful for both atherosclerosis imaging and therapy (32).

Christie Ballantyne, who owes his Greek descent to his mother's family, is an expert on heart disease prevention. He contributed to our understanding of the interplay among lipids, inflammation, and atherosclerosis (33). His early career focused on basic research on the role of endothelial-leukocyte adhesion molecules in inflammatory disease by generating several “knock-out” murine models with target deletions in adhesion molecules (34). He continued to pursue research on inflammation in translational studies of novel biomarkers as the director of the core laboratory of the large NHLBI-funded Atherosclerosis Risk in Community Study which led to FDA approval of 2 biomarkers. He has also played a leading role in clinical trials testing new therapies for lipids and atherosclerosis, ranging from early phase 1 and 2 trials to large outcome trials such as the REDUCE-IT study which showed the benefit of eicosapentaenoic acid in reducing ASCVD events in high-risk patients with elevated triglycerides (35, 36). His work makes him one of the world leaders in innovative medications for atherosclerosis and cardiovascular disease.

Christodoulos I. Stefanadis was trained mostly in Athens but also the US and practiced cardiology primarily as a faculty of the National Kapodistrian University of Athens, Greece. His main research interests included coronary heart disease, detection and treatment of vulnerable atheromatic plaque, aortic elastic properties, mitral valve disease, and interventional treatment of resistant hypertension. His team designed prototype cardiac catheters (37) which were applied in various diagnostic and therapeutic interventions, as well as the technique of retrograde non-transeptal mitral balloon valvuloplasty (38). A large part of his research efforts was devoted to the estimation of the thermal heterogeneity of atherosclerotic plaques in humans. For this, his team used a special catheter with a thermistor tip (39). Also, they developed external non-invasive heating of stented arterial segments and bevacizumab-eluting stent for the inhibition of microvessel growth in unstable atherosclerotic plaques (40). Thus, Christodoulos Stefanadis has been a pioneer in the invention of devices that hold both prognostic and therapeutic value for atherosclerosis.

George D. Dangas is presently a Professor of Medicine and Surgery at the Mount Sinai School of Medicine in New York. He pursued medical and postgraduate studies at the National Kapodistrian University of Athens, Greece, and finished his clinical training at Brown University and the Mount Sinai Hospital. He is an internationally recognized expert in coronary, heart valve, and vascular disease and specializes in minimally invasive interventional treatments with advanced technologies and medical therapy approaches. His research interests include cardiovascular device therapy and its combination with anticlotting medications. To this end, he has coordinated or participated as the main contributor in large clinical trials identifying markers of kidney injury following percutaneous coronary intervention (41) and optimizing the use of oral anticoagulants for the prevention of atrial fibrillation (42) and thromboembolism (GALILEO trial) (43) after successful transcatheter aortic-valve replacement. He has been among the main contributors of the TWILIGHT trial that proposed monotherapy with adenosine diphosphate receptors of subtype P2Y_12_ blocker (ticagrelor) after a minimum period of dual antiplatelet therapy, as a safer treatment for preventing ischemic events with lower incidence of bleeding compared to continuing combination of ticagrelor and aspirin (44). Overall, his work has improved safety of application of medication-based therapies for cardiovascular disease.

Evangelos Michelakis studies pulmonary hypertension and particularly the role of potassium channels in the control of vascular tone in the pulmonary and systemic circulation. The work of his group associated inhibition of phosphodiesterase-5 with therapeutic effects for pulmonary hypertension (45) and right ventricle dysfunction (46), which makes this approach the most common treatment for the disease. Furthermore, his team have associated pulmonary hypertension with metabolic mitochondrial abnormalities in vascular cells (47). Presently, Evangelos Michelakis is a Professor and Vice-Chair of Research in the Department of Medicine at the University of Alberta in Canada.

Alex C Spyropoulos pursued his education and clinical training in the US. Presently, he is a Professor of Medicine at Hofstra, Northwell School of Medicine in New York. His research work was instrumental in the development of the most widely used global venous thromboembolism risk model—IMPROVE VTE—in hospitalized medical patients (48). He was one of the leading investigators in a landmark clinical trial (49), which proved that a no-heparin bridging approach in perioperative settings is safer and as efficacious as a bridging approach, thus simplifying perioperative care globally. Also, this was the first cardiovascular trial in which a placebo comparator was the “experimental” arm. He was the coordinator of the MARINER randomized controlled trial, which established regulatory approval for the direct oral anticoagulant rivaroxaban for extended post-discharge thromboprophylaxis as a new standard of care in hospitalized medically ill patients (50). Thus, Alex Spyropoulos work offers hope for effective treatment of a disease that affects more than 300,000 people/year in the US.

Leonard Kritharides is professor of Medicine at the University of Sydney, Australia. As a physician-scientist, he has pioneered both mechanistic studies and clinical outcomes research in atherosclerosis. He started his training investigating lipoprotein oxidation and the uptake and metabolism of oxidized lipids by macrophages (51, 52). He has studied the regulation of ApoE secretion from macrophages (53, 54), and contributed to work on the synergy between the transporters ABCA1 and ABCG1 in the removal of cellular cholesterol (55) and the role of HDL particle size, rather than composition, as a key determinant of ABCA1-mediated efflux (56). His group identified intra-coronary platelet activation (57), platelet activation in pulmonary embolism, and recently, a unique phenotype of platelet activation in the elderly (58). Therefore, Leonard Kritharides' work has contributed to the understanding of cellular and physiology mechanisms that cause atherosclerosis and embolism.

### Hypertension

Charalambos P. Gavras started his research in Greece, focusing on a special type of hypertension that affects the kidneys. One of the main conclusions of his work at the time was that it would be useful to measure renin in the kidneys of patients. After four years of training in Glasgow, the academic journey of Gavras continued in the USA. Research of the Gavras group has led to numerous valuable findings over the past few decades. His most important contributions include pre-clinical and clinical studies that introduced inhibition of angiotensin receptor (59, 60) and angiotensin-converting enzyme (ACE) (61–65) as effective therapies for the treatment of patients with hypertension or heart failure. His research contributions are considered historical for the field of hypertension that constitutes one of the main causes of heart failure and stroke.

George L. Bakris, professor at the University of Chicago, has numerous publications in the areas of hypertension, diabetic kidney disease, and the progression of nephropathy. His cardiology research has been focused on hypertension and clinical outcome trials, as well as guidelines on kidney disease and diabetes with a focus on cardiovascular risk reduction. His clinical research team has been heavily involved in the development of blood pressure-lowering agents for resistant hypertension and the development of guidelines in this area (66–69). Their most recent work focuses on the interplay of chronic kidney disease and albuminuria with heart failure (FIDELIO-DKD trial) (70), thus expanding the knowledge about cardiorenal interplay in health and disease.

John B. Kostis is professor at Rutgers University's Robert Wood Johnson Medical School, as well as the founding chair of the cardiology department. Involved in studies for the development of more than 140 pharmacological agents and devices, he held leadership positions in large clinical trials that showed the cardioprotective effect of a diuretic (chlorthalidone) in older patients with isolated systolic hypertension (71), in patients with diabetes (72), as well as in patients with HFrEF and HFpEF compared with the Ca^2+^ channel blocker amlodipine and the *α*-receptor blocker doxazosin (ALLHAT trial) (73). He also had a leading role in the OCTAVE trial that showed the superior antihypertensive efficacy of the dual neprilysin and ACE inhibitor (omapatrilat) vs. a sole ACE inhibitor (enalapril) (74). Starting in the late ‘60 s, he contributed to the development of the Doppler Echocardiogram as a key diagnostic tool (75, 76).

## Development of drugs and devices

Scientists of Greek descent were involved in biotechnology of products related to cardiology as early as the beginning of the 1960 s. It was then that George Hatsopoulos, founder of the Thermo Electron Engineering Corporation, conceived the idea of contributing to the efforts of developing an artificial heart by designing a nuclear-powered mechanical heart (Patent US3379191A; inventor Robert J. Harvey, application filed by Thermo Electron Engineering). Today, Thermedics Inc., which acquired the patent of Thermo Electron Corporation for artificial hearts, makes temporary and permanent ventricular assist systems, which prolong survival for thousands of end-stage heart failure patients.

Pindaros “Roy” Vagelos played a key role in the discovery and commercialization of statins. He is considered an influential academic leader and pioneering researcher, as well as one of the most innovative and successful CEOs of the pharmaceutical industry. He researched fatty acid synthesis and lipid metabolism in the ‘60 s and discovered acyl carrier protein (ACP) (77), a key player in this pathway. After joining Merck, he changed the company's research culture and steered it to the development of the first safe inhibitor of hydroxymethylglutaryl-coenzyme A reductase (78), which controls the rate-limiting step for cholesterol synthesis; this led to the development of statins (79), one of the most prescribed classes of drugs in the world. Later, as the CEO of Merck, Vagelos was instrumental in Merck's decision to make Ivermectin freely available to people in Africa and Central America suffering from onchocerciasis (80); in this way, he helped save millions of people from the second-most common cause of infection-induced blindness. Roy Vagelos, who served as the chairman of the Board of Regeneron Pharmaceuticals until 2023, has pioneered reshaping of the drug discovery and development process by investing in basic science research in the pharmaceutical industry. Moreover, he and his wife have been exemplary philanthropists. Part of the wealth that commercialization of his scientific discoveries created has been distributed back to the society through generous donations that support educational programs of excellence, financial aid for students, and buildings in major academic institutes in the US.

Sotirios Tsimikas is professor of Medicine at the University of California San Diego. He is also the Senior Vice President of Global Cardiovascular Development at Ionis Pharmaceuticals. His research and drug development efforts focus on lipoprotein metabolism, oxidized phospholipids, and proteins that promote hyperlipidemia, lipid-driven inflammation, and atherosclerosis (81). He has overseen over 20 Phase I-III trials and directs the third largest cardiovascular franchise in the industry, with 8 active drugs in Phase I-III development, including modulation of Lp(a), ApoC-III, ANGPTL3, Proprotein convertase subtilisin/kexin 9 (PCSK)9, TTR, and AGT using the anti-sense oligonucleotides technology (82–84). Thus, Sotirios Tsimikas is a pioneer in the development of the next generation of drugs for cardiovascular diseases.

John Maraganore, whose family name is the result of misinterpretation of the name that his ancestor wrote in Greek on the immigration documents upon arrival to the U.S., is a pioneer of the RNA-based drug technology. As a biochemist, he invented bivalirudin, a direct-acting thrombin inhibitor (ANGIOMAXTM). As the founding Chief Executive Officer and a member of the Board of Directors at Alnylam Pharmaceuticals, he directed the companýs efforts to advance small interfering RNA (siRNA)-based therapeutics. The effort concluded with the development and approval by the U.S. Food and Drug Administration (FDA) of the first four RNAi therapeutic medicines: ONPATTRO, the first siRNA-based drug approved by the FDA that lowers expression of transthyretin, GIVLAARI, which targets *δ*-aminolevulinate synthase 1 that produces heme, OXLUMO, which suppresses glycolate oxidase, and LEQVIO that inhibits translation of PCSK9, which leads to lowering of LDL-cholesterol and prevention from atherosclerosis.

Sotirios A. Karathanasis had significant contributions as biochemist, innovator and executive leader in the drug development sector. He has held several key positions in the pharmaceutical industry: currently the Chief Science Officer (CSO) at Enveda Biosciences, where he is leading the company's small molecule drug discovery portfolio, he has previously served as the CSO of endocrine and cardiovascular research at Lilly Research Laboratories, Vice President and Head of Biosciences at AstraZeneca, and Director of Cardiovascular Pharmacology at Pfizer Global Research & Development. He has led drug discovery teams with as many as 250 scientists and holds 23 patents. He is the recipient of the Established Investigator Award of the American Heart Association (1985), and the Irvine H. Page Arteriosclerosis Research Award (1983).

## Service to the international cardiovascular scientific community

Physicians of Greek descent have been very active in serving the cardiovascular research community by holding leadership positions in international scientific societies of cardiology and journals issued by these societies.

Panos Vardas was instrumental in founding the European Society of Arrhythmias in 2010–2011 and served as the President of the European Society of Cardiology (2012–2014), as well as the President of the European Heart Rhythm Association (2009–2011). He is the founder of the ESC Office in Brussels (2013) where he serves currently as Chief Strategy Officer of the European Heart Agency. Through his service as a leader of both the University of Crete Cardiology and the European cardiovascular community, he was involved in clinical studies and guidelines for arrhythmias (85–88), and public health (89).

Gerasimos Filippatos has served as the President of the Heart Failure Association (HFA) of the European Society of Cardiology in 2014–2016. As a clinical investigator, he participates in international networks for studies on cardiometabolic and kidney diseases (70, 90).

In the USA, George Bakris was elected President of the American Society of Hypertension, which he served between 2010 and 2012, before the merging with the American Heart Association in 2017. Edward Fisher was the chief editor of the Atherosclerosis, Thrombosis, and Vascular Biology journal of the American Heart Association in 2011–2012. Julia Grapsa is the acting editor-in-chief of the JACC: Case Reports of the American College of Cardiology.

Descendants of the Greek diaspora in Australia have also assumed leadership roles in scientific societies of that part of the world. Leonard Kritharides was the President of the Cardiac Society of Australia and New Zealand (CSANZ) in 2018–2020. As President of the CSANZ, he initiated the preparation of a series of clinical guidelines for cardiologists during the outbreak of the COVID epidemic, which were widely used in Australia (91). Peter Psaltis served as the President of the Australian Atherosclerosis Society between 2020 and 2022.

## Epilogue—future perspectives

Over time, the Greek medical community demonstrated impressive resilience and maintained its close contact with Western medicine, even before the creation of the modern Greek state in the early 19th century. The 200 years that followed the beginning of the Greek War of Independence (1821) saw the creation of Greek clinical and biomedical institutions, the strengthening of the ties between the Greek medical community and pioneering medical institutions in Western countries, as well as a new generation of Greek researchers in the diaspora, who have contributed significantly to the development of Medicine and Cardiology.

The history of cardiology in Greece is paradigmatic for the biomedical sector of the country. Due to an initial lack of resources and equipment, and despite the local interest and manpower, an external impetus was indeed necessary to bring things into motion—more specifically, influences from Germany, France, and the US. From the 1950 s on, Greek cardiology and cardiac surgery have been following closely and assimilating developments in Europe and the US, while occasionally producing original research.

The successes of cardiologists and cardiovascular researchers of Greek descent constitute sources of inspiration for the next generation of physicians and biomedical scientists for both serving as role models and for creating training and employment opportunities of high caliber. Since cardiology was developed as a separate medical specialty primarily after the Second World War and the subsequent Civil War of Greece, its progression has reflected the country's development. During these years, several physicians and researchers of this specialty have pursued remarkable careers abroad while many of them maintained their ties with the homeland. Beyond training of young physicians and bioscientists in their groups that occurs routinely, their connection and passion about supporting research and education in Greece has translated to international educational programs that facilitated training of the next generation of clinical and basic scientists abroad. The “medical bridge” between the University of Utah and the University of Athens that was established by Ioannis Nanas and the medical student exchange program between the University of Crete and Boston University that was created by Vassilis Zannis represent such programs. These initiatives have served as bidirectional channels of talent flow that create opportunities for young scientists to train abroad and then return, thus contributing to the implementation of international standards in clinical practice and research of Greece. The experience from such programs can be used for the creation of national training programs that would allow cardiology and cardiac surgery fellows of Greek hospitals or graduate students and postdoctoral fellows that pursue cardiovascular research to do part of their training in medical centers and research institutes abroad.

The international Greek example in cardiology and cardiovascular research could have served as a springboard for Greece to capitalize on the intellectual capital of Greek investigators and clinicians and become a pioneering country in clinical and basic cardiology research. Despite the wealth of cardiovascular clinicians and researchers in the country and abroad and their significant achievements, a cardiovascular research institute of international reputation is yet to be created in Greece. Establishment of such an entity that would bring together prominent basic cardiovascular researchers and clinicians and connect them with pharmaceutical industry pioneers would facilitate discovery and development of innovative drugs and therapies. This would constitute a hub of excellence in cardiovascular research that would create training and employment opportunities for the brightest of the country's youth and would contribute to the growth of the economy.

This paper has mentioned some of the most prominent representatives of cardiology, cardiac surgery, and cardiovascular research of Greek descent. The authors are aware that inclusion of all would have been *a priori* impossible. Furthermore, as this history of Greeks in modern cardiology is being written, there is already a new and growing generation of clinical and biomedical researchers rising, both in Greece and abroad. They are already contributing impressively, and they will certainly have their position in future studies about fundamental contributions of Greeks in cardiology, cardiac surgery and cardiovascular research.
