# Corrigendum: 1821–2021: contributions of physicians and researchers of Greek descent in the advancement of clinical and experimental cardiology and cardiac surgery

**DOI:** 10.3389/fcvm.2023.1282686

**Published:** 2023-09-07

**Authors:** Apostolos Gerontas, Dimitrios Avgerinos, Konstantinos Charitakis, Helena Maragou, Konstantinos Drosatos

**Affiliations:** ^1^ School of Applied Natural Sciences, Coburg University, Coburg, Germany; ^2^ School of Liberal Arts and Sciences, The American College of Greece, Athens, Greece; ^3^ Department of Cardiac Surgery, Onassis Cardiac Surgery Center, Athens, Greece; ^4^ ARISTEiA-Institute for the Advancement of Research and Education in Arts, Sciences and Technology, McLean, VA, United States; ^5^ Department of Internal Medicine, Division of Cardiology, University of Texas Health Science Center, Houston, TX, United States; ^6^ Metabolic Biology Laboratory, Cardiovascular Center, Department of Pharmacology and Systems Physiology, University of Cincinnati College of Medicine, Cincinnati, OH, United States

**Keywords:** Greece, cardiology, cardiac surgery, cardiovascular research, pioneers, experimental cardiology, physicians, researchers

A Corrigendum on 1821-2021: contributions of physicians and researchers of Greek descent in the advancement of clinical and experimental cardiology and cardiac surgery By Gerontas A, Avgerinos D, Charitakis K, Maragou H and Drosatos K. (2023) Front. Cardiovasc. Med. 10:1231762. doi: 10.3389/fcvm.2023.1231762

**Error in Figure**

In the published article, there was an error and omissions in **Figure 1** as published. Information regarding the 1st Department of Cardiology at the AHEPA Hospital of the Aristotle University of Thessaloniki was inadvertently omitted. The Department of Cardiology was founded by Dimitrios Tsifodimos in 1986. The founding chair of the Cardiac Surgery Department of the Aristotle University of Thessaloniki, Dimitrios Lazaridis, was also the founding director of the Cardiac Surgery Department of the Hippokration Hospital of the University of Athens in 1962. In 1965, Georgios Andritsakis, who is mistakenly mentioned in the original article as the founding director of the Department of Cardiac Surgery at the Hippokration Hospital, succeeded Dimitrios Lazaridis. Also, it was inadvertently not mentioned that Georgios Tolis performed the first coronary bypass operation in Greece in 1971. Furthermore, Stefanos Roussis, Georgios Louridas, and Georgios Giannoglou performed the first percutaneous coronary angioplasties in Greece in 1985 at the Papanikolaou Hospital and the AHEPA Hospital of Thessaloniki.

**Figure 1:**
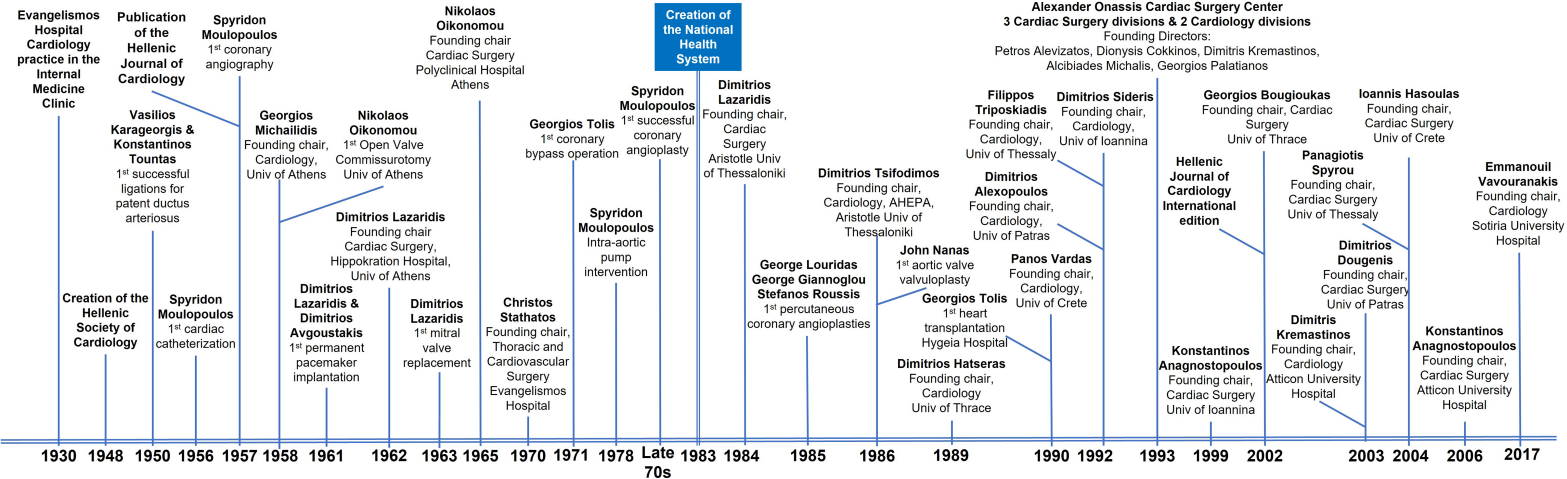
Important events and contributors in the development of cardiology and cardiac surgery in Greece.

The corrected **Figure 1** and its caption appear below.

**Text Corrections**

A correction has been made to **the CARDIAC SURGERY IN GREECE section**, Paragraph two.

This sentence previously stated:

“in 1965, Georgios Andritsakis founded the first dedicated cardiac surgery unit at the Hippokration hospital in Athens.”

The corrected sentence appears below:

“in 1962, Dimitrios Lazaridis founded the first dedicated cardiac surgery unit at the Hippokration hospital in Athens and was succeeded by Georgios Andritsakis in 1965”

An addition has been made to correct an omission in **the CARDIAC SURGERY IN GREECE section**, Paragraph three.

The added sentence appears below:

“Georgios Tolis performed the first coronary bypass operation in Greece in 1971.”

An addition has been made to correct an omission in **the CARDIAC SURGERY IN GREECE section**, Paragraph five.

The added sentence appears below:

“In 1985, Stefanos Roussis, Georgios Louridas, and Georgios Giannoglou performed the first percutaneous coronary angioplasties in Greece at the Papanikolaou Hospital and the AHEPA Hospital of Thessaloniki.”

The authors apologize for these errors and state that these do not change the scientific conclusions of the article in any way. The original article has been updated.

